# Real-Life Report on Chemoembolization Using DEBIRI for Liver Metastases from Colorectal Cancer

**DOI:** 10.1155/2015/715102

**Published:** 2015-02-26

**Authors:** M. Stutz, A. Mamo, D. Valenti, A. Hausvater, T. Cabrera, P. Metrakos, P. Chaudhury, G. Steacy, E. Garoufalis, P. Kavan

**Affiliations:** ^1^Segal Cancer Centre, JGH, McGill University, Canada; ^2^Radiology, McGill University Health Centre, Canada; ^3^Hepatobiliary Surgery, McGill University Health Centre, Canada; ^4^Oncology, McGill University Health Centre, Canada

## Abstract

*Background*. Transarterial chemoembolization (TACE) has been investigated in patients with liver metastases from colorectal cancer (LMCRC). Limited experience and available data suggest that TACE can achieve disease stabilization or improvement, even in heavily pretreated patients. *Methods*. Patients with LMCRC, ECOG 0–2, who failed at least 1 line of systemic chemotherapy, received embolizations with 2 mL of microspheres preloaded with 100 mg of irinotecan. Beads were delivered selectively into hepatic arteries. Primary endpoint was overall survival (OS), analyzed using the Kaplan-Meier method. Secondary endpoint was safety, assessed using CTCAE version 4.0. *Results*. 27 patients were treated using DEBIRI. Patient median age was 57 years (range was 45–82 years). The median number of total embolizations was 1.3 (range 1–3). The median OS was 5.4 months (95% CI; 1.1–22.7 months). The most reported postembolization events were nausea (8/27), vomiting (6/27), right upper quadrant pain (16/27), fatigue (9/27), and the development of ascites (6/27). 5/26 patients required hospitalization after TACE for severe pain. Hospitalization was also required for 1 case of allergic reaction and 1 case of infection. *Conclusion*. Our data suggest that TACE with DEBIRI could be efficacious in a palliative setting for patients with LMCRC, but they do not necessarily support routine use in clinical practice.

## 1. Introduction

Colorectal cancer (CRC) remains one of the leading causes of cancer-related deaths worldwide. Synchronous or metachronous liver metastases can be present in almost half of all individuals diagnosed with CRC [1]. Although the gold standard for treatment of liver metastases has classically been liver resection, only 10–15% present with resectable tumors; consequently an inoperable tumor is associated with poor prognosis [2]. Furthermore, in individuals with resected liver metastases, the 5-year survival rate is 25–37% and of this group 70–80% will have a relapse of which approximately half will reoccur in the liver [2, 3]. Several locoregional intervention therapies exist, aiming at further increasing survival, resectability, and quality of life. One such locoregional therapy is transarterial chemoembolization (TACE) [4].

TACE takes advantage of the fact that metastatic tumours in the liver derive their main blood supply almost completely from the hepatic artery (HA). On the other hand, the normal liver derives only 30% of the blood supply from the HA and instead relies predominantly on the portal vein. TACE prevents blood supply from reaching the tumor through the angiographic insertion of a catheter loaded with an embolizing material that inhibits blood flow of selectively targeted branches of the HA. Combined with the simultaneous infusion of a chemotherapeutic agent(s), TACE leads to tumour ischemia and necrosis. Additionally, it enables the targeted drug delivery while extending drug exposure time by reducing the blood flow [3]. A number of embolizing and chemotherapeutic agents have been introduced in clinical practice in recent times, including irinotecan-loaded drug-eluting beads (DEBIRI). DEBIRI is composed of permanent microspheres that bind irinotecan and release the drug over time at the tumour site [5].

As a neoadjuvant tool for downstaging unresectable metastatic tumour burden to the liver, Martin et al., in a multi-institutional, single arm pilot study, demonstrated that 20% of individuals TACE'd with DEBIRI experienced either significant response with disease downstaging or stable disease that permitted resection and/or radiofrequency ablation (RFA) [6]. Similarly, in an interim report on a phase II study TACE using DEBIRI for the treatment of MCRC, Fiorentini et al. reported that 10% of individuals experienced downstaging that led to resection or RFA [7, 8]. This similar study also investigated TACE using DEBIRI as a second- and third-line treatment. It was found to significantly benefit heavily pretreated individuals who had failed first- and second-line chemotherapy, improving both PFS (11 months) and median overall survival (OS) (19 months), as well as response rates (65%, 50%, and 40% at 3, 6, and 12 months, resp.) when compared to third-line chemotherapy regimens [9–11]. Furthermore, Martin et al. identified that the extent of prior chemotherapy, as well as the presence of extrahepatic metastases, was predictors of OS [6].

In a phase III study by Fiorentini et al., they assessed DEBIRI in the treatment of LMCRC; comparison of DEBIRI to systemic FOLFIRI was performed in 74 patients refractory to 2-3 lines of systemic chemotherapy. Patients were randomized to receive either two cycles of DEBIRI or 8 cycles of FOLFIRI. Overall, the study found that DEBIRI was superior to systemic FOLFIRI: median survival was 22 months versus 18 months, PFS was 7 months versus 4 months, time to extrahepatic progression (TEP) was 13 months versus 9 months (although statistically not significant), objective response rates were 68.6% versus 20%, and DQoL was 8 months versus 3 months, respectively [12].

This retrospective study reports a single institution's experience of TACE for metastatic colorectal cancer (MCRC) patients with unresectable liver metastases in a real-life setting.

## 2. Materials and Methods

A retrospective analysis of 27 patients with histologically confirmed CRC and unresectable liver metastasis was undertaken from 2011 to 2013. All patients were refractory to at least one line of systemic chemotherapy (average 2; range 1–5), received at least one treatment of DEBIRI TACE (average 1.3; range 1–3), and had an ECOG ≤ 2 at first TACE.

### 2.1. DEBIRI TACE Procedure

DEBIRI was created by loading DC beads with 50 mg irinotecan per 100 mL microspheres, with a maximum of 200 mg irinotecan loaded onto 4 mL microspheres, in a 2-hour loading period. DEBIRI administration was done using angiography, and catheter was selectively placed where the maximum blockage of blood flow to the tumour was achieved. Access to the common HA was gained via the perfusion of the common right femoral artery, and embolization was performed in a segmental or lobar fashion, depending on the distribution and extent of disease. Repeat of the procedure was at the discretion of the physician. All patients were treated according to standard hospital procedure for TACE; routine prophylactic treatments against nausea and vomiting, infection, and upper right quadrant pain were also given to patients prior to the procedure. These include antiemetic medications as well as antibiotics and are standard treatment given prior to TACE procedures. Response rates were evaluated using CT scans 1-2 months after procedure and then every 3 months or when clinically indicated. Results were read by the treating physician and an independent investigator using RECIST 1.1 criteria.

### 2.2. Statistical Analysis

The primary endpoint of this analysis was safety. Grading of the adverse events was determined using CTCAE version 4. Secondary endpoint of the study was OS, defined as time from the last TACE treatment with DEBIRI to death. The Kaplan-Meier method was used to analyze survival data.

## 3. Results

### 3.1. Patient and Treatment Characteristics

Twenty-seven patients with LMCRC underwent a total of 36 treatments of DEBIRI at the McGill University Hospital Center. Median age of patients was 57 years (45–82 years, 16/27 males). At the time of embolization, 40% of patients presented with extrahepatic metastases. Of the 27 patients, 15 (55%) had had prior hepatic surgery (hepatic lobectomy, PVE, RFA, or a combination of the three). A majority (56%) of patients had an ECOG = 0. Significant medical history included cardiac, diabetes, hypertension, and previous cancers (Table 1).

On average, patients had at least 2 lines of chemotherapy (range 1–5). Eight out of twenty-seven patients were refractory to only one line of systemic chemotherapy. For patients who failed first-line chemotherapy, proportions were as follows: FOLFOX ± bevacizumab (21/27; 78%); FOLFIRI ± bevacizumab (5/27; 18.5%); and FOLFIRINOX + bevacizumab (1/27; 3.5%). For those who failed second-line therapies, proportions were as follows: FOLFOX ± bevacizumab (5/19; 26%); FOLFIRI ± bevacizumab (12/19; 63%); and 2/19 (10%) were treated with FOLFIRI + pentamidine or XELIRI + cetuximab. For those who failed third- and fourth-line therapies, we obtained the following: FOLFIRI (3 patients), FOLFOX (3 patients), FOLFIRINOX (1 patient), XELODA (1 patient), IROX (1 patient), and pentamidine (1 patient), as well as 6 patients receiving various biologics (Table 1).

All patients received at least one session of TACE, whereas 20 patients (74%) received only 1 treatment, 5 patients (18.5%) received 2 treatments, and 2 patients (1.5%) received 3 treatments, with an average of 1.3 per patient (Table 4). All patients were treated with DC beads 100 *μ*m–300 *μ*m in size, and 100 mg of irinotecan was administered in 34/36 procedures. Two procedures with 200 mg of irinotecan were performed in two different individuals. The right lobe was the most frequent target (21/36; 58.5%), and one bronchial artery embolization was performed (Table 4). No complications were reported during the procedure. After embolization, 7 patients continued systemic therapy (Table 2).

### 3.2. Safety

Most patients experienced symptoms of postembolization syndrome (PES). The most common adverse events (AEs) were as follows: vomiting (6/26; 22%); nausea (8/27; 30%); fatigue (9/27; 33%); development of ascites (6/27; 22%). Some of these symptoms required hospitalization: 5 cases of right upper quadrant pain as well as one case of infection and allergic reaction in a patient who had already received three DEBIRI treatments (Table 3).

### 3.3. Efficacy

**Figure 1:**
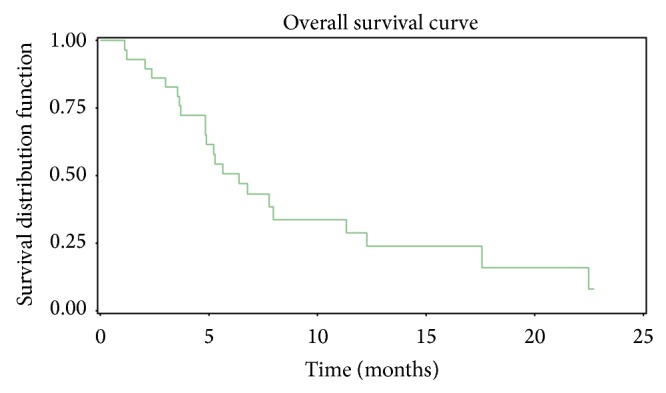


Median OS (defined as time from last treatment to death) was found to be 5.4 months (95% CI; 1.1–22.7 months) one year after the last treatment of chemoembolization (Figure 1). Predictors of OS at the time of embolization were based primarily on those that have been mentioned in the literature [6, 12]. Although extremely preliminary due to small number of patients, we divided 27 patients into 3 categories as follows: patients still alive (*N* = 6), patients whose OS was less than 5 months (*N* = 9), and patients whose OS exceeded 5 months (*N* = 12). The arbitrary assignment of 5 months as the cut-off point was based on median OS. The amount of total TACE, previous lines of chemotherapy, ECOG status at time of embolization, and presence of extrahepatic metastases (Table 4), as well as previous portal vein embolization (PVE), RFA, and hepatic lobectomies (Table 5) were reported. The mean total amount of TACE for the alive and OS > 5 months (mo) and OS < 5 mo subgroups was 1.5, 1.3, and 1.0, respectively; the mean number of previous chemotherapy lines for the alive and OS > 5 mo and OS < 5 mo subgroups was 1.8, 1.8, and 2.5, respectively; the percent of individuals with an ECOG status of 0 at the time of embolization for the alive and OS > 5 mo and OS < 5 mo subgroups was 66%, 67%, and 22%, respectively; and the percent of extrahepatic metastases in the alive and OS > 5 mo and OS < 5 mo subgroups was 50%, 34%, and 44%, respectively. Notably, the majority of PVEs, RFAs, and hepatic lobectomies (86%, 75%, and 64%, resp.) were performed in the alive and OS > 5 mo combined subgroup. Hospitalization after treatment was recorded for all subgroups. Seven out of eight patients (87%) with OS > 5 mo were hospitalized following chemoembolization whereas only 1 patient in the OS < 5 mo subgroup was hospitalized (Table 5).

## 4. Discussion

In this retrospective analysis, we report a median OS of 5.4 months much lower than 22 months reported by Fiorentini et al. [7, 8, 12] who looked at DEBIRI versus systemic FOLFIRI in individuals who were refractory to 2-3 lines of systemic chemotherapy and had unresectable LMCRC involving <50% of the liver, no extrahepatic metastases, no history of past cancer, no previous RFA or PVE, and bilirubin ≤ 2 times the upper limits of normal and whose last chemotherapy treatment was > 3 months prior to embolization. From this, one can argue that the study by Fiorentini et al. is an example of DEBIRI treatment optimization, highlighting the importance of patient selection. Our retrospective report included heavily pretreated individuals in real-life practice with less stringent selection criteria. In fact, our preliminary data showed that previous lines of chemotherapy and ECOG performance status at the time of embolization were associated with longer median OS, where the average number of failed chemotherapy lines prior to treatment was the highest in the OS < 5 mo subgroup (2.5) and lower in the alive and OS > 5 mo subgroups (both at 1.8). Also, only 22% of individuals in the OS < 5 mo subgroup had an ECOG PS of 0 prior to treatment, whereas 66% and 67% of individuals in the alive and OS > 5 mo subgroups had an ECOG PS of 0. Therefore, our data reinforced the previous work that has linked limited previous chemotherapy and low ECOG PS at time of embolization to better outcome [7, 8, 12, 13]. No correlation between the presence of extrahepatic metastases and survival [7, 8, 12] was found in our report due to limited number of patients. Interestingly, a study by Huppert et al. showed that median OS was longer in patients with limited (<25%) compared with extensive (>50%) intrahepatic disease (21 versus 5 months, *P* < 0.005) [14]. Moreover, Narayanan et al. showed that DEBIRI is a well-tolerated treatment option that can be used safely in the palliative treatment of hepatic metastases from CRC, with a median OS from first DEBIRI treatment being 13.3 months (95% CI 6.8–19.8 months) [15].

An interesting finding observed in our study was the prevalence in both the alive and OS > 5 mo subgroups, of individuals who had undergone a prior hepatic surgery. In fact, disease control through resection or ablation may enhance DEBIRI synergistically, and PVE (which involves hepatic hypertrophy through vascular occlusion 17) may be useful in preparing the liver for procedures other than hepatic lobectomy. Safety analyses were similar to previous reports [7, 8, 12]. Notably, a few studies have addressed the role of DEBIRI in LMCRC. Martin et al. assessed the safety, tolerance, and pharmacokinetic profile of DEBIRI in combination with FOLFOX ± bevacizumab in 10 treatment-naive individuals with unresectable LMCRC. Twelve cycles of FOLFOX and at least 2 DC beads treatments were administered. This phase I study debuts the use of DEBIRI in a first-line setting and in combination with systemic chemotherapy. It was found that not only was DEBIRI with concomitant FOLFOX ± bevacizumab safe and well tolerated with limited AEs reported but it also enhanced overall response rate; at 9 and 12 months, 100% of individuals were still benefiting from the treatment, and 4 patients successfully had their disease downstaged to resection 18. Another clinical trial currently underway addresses the use of DEBIRI in combination with cetuximab (DEBIRITUX) versus systemic chemotherapy with cetuximab and FOLFIRI, in patients with refractory MCRC. This is a phase II trial, whose primary endpoint is the rate of PFS after 6 months, where safety, tumour response, time to progression (TTP), and OS constitute some of the secondary endpoints 18. The two abovementioned trials are representative of the shift occurring regarding the use of DEBIRI in the treatment of unresectable LMCRC; moving away from a palliative setting, DEBIRI is being explored as an addition to systemic chemotherapeutic treatment regimens. As liver metastasis is one of the main causes of mortality in CRC patients, local disease control provided by DEBIRI can well complement the cytotoxic action of systemic therapy.

Our data suggests that TACE with DEBIRI could be efficacious in a palliative setting for patients with LMCRC. Nevertheless, phase III randomized trials are needed in order to determine how to best optimize the timing of this procedure in an appropriately selected patient base. We therefore propose that identifying potential predictive factors for the treatment of LMCRC with DEBIRI is warranted for better outcomes to understand efficacy and safety. In the meantime, DEBIRI routine use in unselected patient population remains uncertain outside the clinical trial.

## Figures and Tables

**Table 1:** Patient characteristics.

Patient characteristics		
Median age, years (range)	57, (45–82)	

	Patient number; *N* = 27	Percent

Gender (M/F)	16/11	60/40
Prior hepatic procedure		
PVE	1	3.5
H. lobectomy	4	15
PVE + h. lobectomy	2	7.5
PVE + RFA	1	3.5
RFA + h. lobectomy	4	15
PVE + RFA + h. lobectomy	3	11
Total	**15**	**55.5**
ECOG performance status	*N* = 25	
0	14	56
1	9	36
2	2	8
Extrahepatic disease (Y/N)	11/16	40/60

Previous medical history	Number of patients; *N* = 27	Percent

Diabetes	4	15
Cardiac	4	15
Hypertension	12	45
Previous cancer	3	11
Breast	2	7.5
Prostate	1	3.5

Previous chemotherapy		

Number of previous lines failed	Number of patients; *N* = 27	Percent

1	8	30
2	12	44
3	4	15
4	2	7
5	1	4
Average chemotherapy lines/patient	2	
First line	*N* = 27	
FOLFOX ± bevacizumab (+/−)	21 (17/4)	78
FOLFIRI ± bevacizumab (+/−)	5 (3/2)	18.5
FOLFIRINOX ± bevacizumab	1	3.5
Second line	*N* = 19	
FOLFOX ± bevacizumab (+/−)	5 (4/1)	26
FOLFIRI ± bevacizumab	12 (9/3)	64
FOLFIRI + pentamidine	1	5
XELIRI + cetuximab	1	5
Postembolization treatment		
Chemotherapy	7	26
Panitumumab	4	15
Total	**11**	**41**

**Table 2:** Characteristics of treatment DEB-TACE.

Treatment (tx) characteristics		
Number of embolizations	Patient number; *N* = 27	Percent

1	20	74
2	5	18.5
3	2	1.5
DC bead size 100–300 *μ*m	27	100
Technical success	27	100
Total embolizations performed	36	
Average embolization/patient	1.3	

Tx location	Number of tx; *N* = 36	Percent

Right lobe	21	58.5
Left lobe	13	36
Both	2	5.5
Bronchial	1	2.5

Dose delivered	Number of tx; *N* = 36	Percent

100 mg	34	94.5
200 mg	2	5.5

**Table 3:** Adverse event after DEB-TACE.

AE	All grades number of events	Severe grade number of events	Percent event (all grades); *N* = 27
Nausea	8	0	30
Vomiting	6	0	22
Fatigue	9	0	33
Ascites	6	1 (16.5%)	22
Anorexia	2	0	7.5
HTN	1	0	3.5
Skin rash	1	0	3.5
Pain	16	5 (31.5%)	60
Infection	1	1 (100%)	3.5
Allergic rxn.	1	1 (100%)	3.5

Total	51	8	

**Table 4:** Sites of disease progression after DEB-TACE.

Location of disease progression	Number of patients; *N* = 27	Percent
Liver	21	78
Lungs	2	7.5
Both	3	11
Died before progressing	1	3.5

**Table 5:** Subgroup analysis after DEB-TACE.

Alive	Total embolizations	Number; percent	Previous failed chemotherapy	Number; percent	ECOG	Number; percent	Extrahepatic metastases	Number; percent	QoL	Number; percent	SAEs [*N* = 8]	Percentage
*N* = 6	1 2 3	4; 66% 1; 16% 1; 16% Mean = 1.5	1 2 3 4 5	2; 34% 3; 50% 1; 16% 0 0 Mean = 1.8	0 1 2 n/a	4; 66% 1; 16% 1; 16% 0 Mean = 0.5	Y N	3; 50% 3; 50%	Stable Delayed Immediate n/a	3; 50% 1; 16% 0 2; 34%	0	0

OS < 5 months	Total embolizations	Number; percent	Previous failed chemotherapy	Number; percent	ECOG		Extrahepatic metastases	Number; percent	QoL	Number; percent	SAEs *N* = 8	SAEs *N* = 8

*N* = 12	1 2 3	7; 58% 4; 33% 1; 9% Mean = 1.3	1 2 3 4 5	5; 41% 5; 41% 1; 9% 1; 9% 0 Mean = 1.8	0 1 2 n/a	8; 67% 2; 16% 0 2; 16% Mean = 0.15	Y N	4; 34% 8; 66%	Stable Delayed Immediate n/a	7; 58% 2; 16% 2; 16% 1; 9%	7	87.5%

OS < 5 months	Total embolizations	Number; percent	Previous failed chemotherapy	Number; percent	ECOG		Extrahepatic metastases	Number; percent	QoL	Number; percent	SAEs *N* = 8	SAEs *N* = 8

*N* = 9	1 2 3	9; 100% 0 0 Mean = 1.0	1 2 3 4 5	1; 11% 5; 55% 1; 11% 1; 11% 1; 11% Mean = 2.5	0 1 2 n/a	2; 22% 6; 66% 1; 11% 0 Mean = 0.9	Y N	4; 44% 5; 56%	Stable Delayed Immediate n/a	0 2; 22% 6; 67% 1; 11%	1	12.5%
